# Web-Based Interventions Reduced Dental Anxiety among Adults in Lithuania and Norway: A Pilot Study

**DOI:** 10.3390/ijerph20043343

**Published:** 2023-02-14

**Authors:** Lina Stangvaltaite-Mouhat, Indre Stankeviciene, Sigrid Sofia Sanchez Martinussen, Vytautas Sabataitis, Camilla Sandjord, Ingrid Toresen, Marianne Stoltenberg Tryggestad, Alina Puriene, Jan-Are Kolset Johnsen

**Affiliations:** 1 Oral Health Centre of Expertise in Eastern Norway, 0369 Oslo, Norway; 2 Institute of Dentistry, Faculty of Medicine, Vilnius University, 03101 Vilnius, Lithuania; 3 Department of Clinical Dentistry, Faculty of Health Sciences, UiT The Arctic University of Norway, 9037 Tromsoe, Norway

**Keywords:** adult, community dentistry, dental anxiety, digital technology, mental health, oral health self-efficacy, public health

## Abstract

Dental anxiety (DA) is a prevalent public health issue. However, there is a lack of self-administered DA interventions. The aim of this study was to evaluate the short-term effects of web-based interventions aiming to reduce DA in adults in two European countries. A pretest posttest design was used. Tailor-made websites were developed in Lithuania and Norway. Volunteers who self-reported DA were invited to participate. DA levels measured by the Modified Dental Anxiety Scale (MDAS) were assessed at baseline and after two weeks via online questionnaires. The interventions were completed by 34 participants in Lithuania and 35 participants in Norway. In Lithuania, the median posttest MDAS score (9.5, IQR 5.25) decreased compared to the median pretest MDAS score (14.5, IQR 8; Z value = −4.246, *p* < 0.001). The same was found in Norway—the median posttest MDAS score (12, IQR 9) was lower compared to the median pretest MDAS score (15, IQR 7; Z value = −3.818, *p* < 0.001). The present study demonstrated that two tailor-made web-based interventions had the potential to reduce dental anxiety levels when assessed in the short term in Lithuania and Norway. Studies with more controlled designs assessing long-term outcomes are needed to validate the results of this pilot study also in other cultures.

## 1. Introduction

Dental anxiety (DA) is a public health issue [1] as DA, in varying degrees, impacts oral health. For instance, patients’ oral health status, oral health-related quality of life, self-esteem, and oral health behaviors [2,3,4,5,6]. Globally, DA among adults is prevalent [7]. In Norway, as well as in Western Europe, it is stipulated that about 5% of the population suffers from the most severe form of DA—the so-called dental phobia [8,9]. In Lithuania, the prevalence of DA among adults is unknown. A recent study showed that 68% of 15–18-year-old adolescents reported being afraid of or concerned about dental treatment, and out of them, 13% had severe DA [2].

Although DA is sometimes discussed in general terms as a unified phenomenon, people with DA are not homogenous, and their anxiety can be specified as more or less severe based on the severity and nature of the symptoms experienced by the individual [10]. While the less severe forms of DA are perhaps experienced as discomfort or trepidation about dental visits and procedures, the most severe forms of DA are referred to as “dental phobia”. Dental phobia is commonly regarded as a subgenre of specific phobias where one of the key defining aspects is the avoidance of dental treatment [11]. Thus, for sufferers of dental phobia, the consequence of avoiding dental treatment or check-ups could be the deterioration of oral health status both in the short and long term [12,13]. Recent years have seen an increased interest in dental phobia as a threat to oral health, which has renewed interest in understanding the causes of severe forms of DA, as well as investigating effective treatment programs. In Norway, treatment of dental phobia is performed in accordance with a treatment manual approved by the Norwegian Directorate of Health and within the public treatment program known as TADA (torture, abuse, and dental anxiety) [14]. TADA is offered for individuals that are diagnosed with dental phobia and also for individuals that have suffered dental health issues after being victims of abuse or torture. In Lithuania, presently, there are no interventions targeting any forms of DA, except dental treatment under general anesthesia for children with severe DA [15].

While Cognitive Behavioral Therapy (CBT) has been shown to be beneficial for patients with severe dental anxiety and dental phobia, it is nevertheless a treatment form that requires substantial public resources when implemented at the population level, for example, as in the Norwegian TADA initiative. It is thought-provoking that people with low and moderate DA levels do not have any offers targeting their problems, which could arguably be useful to prevent DA from worsening. However, the nature of an intervention targeting less severe dental anxiety would likely differ from the therapeutic approaches targeting severe DA and dental phobia. For instance, patients with moderate levels of DA might have different needs than sufferers of severe DA, and most people in this group would probably not see themselves as patients requiring psychological treatment such as CBT, perhaps due to the stigmatization surrounding mental illness [16]. Therefore, DA interventions that have a broad public health perspective could be of value in Lithuania and Norway.

One key premise of web-based interventions containing oral health/DA information and advice for patients with DA is that they would have an effect by improving knowledge of these topics and associated behaviors in such a way that it would strengthen individuals’ beliefs in their capacity to cope with their anxiety, i.e., increase their self-efficacy for this particular topic. For instance, higher knowledge levels are found to be associated with problem-oriented coping, self-management, and self-efficacy among diverse patient groups [17,18,19]. Self-efficacy is defined as the expectations or beliefs individuals have about their ability to perform a behavior [20], and it has been shown that general self-efficacy is associated with lower levels of general anxiety [21] and dental anxiety [22]. Though general self-efficacy does not always appear related to DA [23,24], specific types of self-efficacy, e.g., “oral health self-efficacy,” might be more reliably associated with DA, as this concept may prevent and reduce DA for some people [21]. However, little data is available regarding oral health self-efficacy (OHSE) and DA. OHSE is perhaps the most relevant when thinking about coping with less severe forms of DA, and it would be reasonable to investigate if online interventions targeting DA and providing oral health information could increase OHSE and reduce DA in adults.

The aim of this study was to design self-administered web-based interventions aiming to reduce DA in adults and evaluate their short-term effects in two European countries, namely Lithuania and Norway. We also investigated what effects these web-based interventions had on participants’ OHSE.

## 2. Materials and Methods

### 2.1. Website

Two different tailor-made websites were developed in Lithuania and Norway, which were used as an intervention in each country (Table 1 and Table 2). The design and development of web-based interventions were performed in 2019, and data collection took place in 2020–2021. The rationale for designing self-administered web-based interventions is presented below.

Increasing use of online services providing information about health and dentistry has been reported [25,26]. Accessing health information on the internet allows anonymity, flexibility, and accessibility and could ultimately be cost-effective [27]. While dentists appear cautiously optimistic about the potential of using technology to expand access to dental healthcare services [28], technology-based interventions have been shown to be successfully integrated into dentistry by improving oral health knowledge, attitudes, and oral health self-efficacy [29,30]. The World Health Organization, through the recently adopted Oral Health Resolution and the Draft Global Oral Health Action Plan, calls to tailor digital interventions addressing oral health along the life course with special consideration among others for people in disadvantaged situations [31,32], such as those having DA. When targeting anxiety related to medical procedures, digital technologies can be used to prepare patients for a medical procedure by providing information (preoperative preparation) or by distraction during a medical procedure [33]. A systematic review found that technology-based preoperative preparation interventions had the potential to reduce anxiety in children and parents, though it was difficult to conclude which intervention was the best technology-based intervention [34]. Another systematic review related to children and adults with moderate and severe DA indicated that technology-based interventions, such as video modeling, computerized cognitive behavior therapy, virtual reality exposure therapy, and distraction with audiovisual video, seem to be promising for reducing DA [35]. Similarly, a recent systematic review found that the use of virtual reality interventions as a distraction or preoperative preparation tool had the potential to reduce DA in children [36]. Also, internet-based resources have been included in self-help programs aimed at children’s DA [37]. Concerning adults, a randomized controlled trial showed that virtual reality-induced relaxation reduced preoperative DA [38]. The above-mentioned technology-based interventions require some degree of health personnel involvement since they are completely or partly administered in a clinical setting. Self-administered interventions could potentially save recourses while providing patients with knowledge about specific topics related to DA. A study reviewing existing websites about dental extractions and DA that could serve as a self-administered preoperative patient tool found that the educational value of the available websites were low, with them lacking information about the procedure itself, anesthesia, and methods for relaxation [39]. A recent randomized controlled trial showed to reduce post-operative levels of DA in patients with transcrestal sinus floor elevation who used a preoperative online intervention compared to a non-online intervention [40]. The authors concluded that online intervention improved patients’ understanding of the procedure and resulted in reduced DA.

For the web-based interventions’ websites in Lithuania and Norway, the cultural differences between the countries will naturally condition the design and implementation process [41]. The Legatum Prosperity Index (LPI) is an annual ranking of countries and territories, which reflects the overall quality of life and human development level by assessing 104 variables in 12 categories [42]. According to the 2019 LPI, Norway was ranked as the second country overall, with specific rankings in Education—11, in Health—5, and in Living Conditions—7. In overall ranking, Lithuania was 33; in Education—27, in Health—92, and in Living Conditions—38.

Therefore, the research hypotheses of this pilot study were as follows. Hypothesis 1: Participating in the tailor-made web-based intervention would reduce participants’ DA. Hypothesis 2: Since the web-based interventions are conditioned by the culture in which they were designed and implemented, we expected differences between Norway and Lithuania with regard to how the interventions change the participants’ beliefs about their own ability to cope with their oral health concerns as measured by OHSE).

#### 2.1.1. Lithuania

The tailor-made website section targeting individuals with DA was named “Ne(Bijau)” (translation “Not(afraid)”) and was created as a part of a larger website for public education in oral health (“https://www.virtualusodontologas.lt/ (accessed on 13 February 2023)”). The website section consisted of six modules containing articles. The first module presented an article about DA and advice on how to manage it during dental procedures [43,44]. The other two modules focused on basic knowledge about dental diseases and their prevention. The last three modules contained articles that detailed the most common clinical procedures, namely professional oral hygiene, placing a filling, and root canal treatment. Each of the modules contained visual aids and links to other relevant information on the same website or other sources (e.g., videos of procedures on YouTube). At the end of each module, homework tasks were presented, which encouraged reflection on the presented information, remembering previously provided information, and/or how to apply it practically.

#### 2.1.2. Norway

A tailor-made website, “Tannvett” (translation “ToothSense” was developed under the domain of UiT—The Arctic University of Norway (UiT; “https://uit.no/research/tannvett (accessed on 13 February 2023)”). The development was inspired by prior studies of health-related web-based interventions [29,30], and a consultant from the TADA program was asked to provide input during the development. The information was presented in several ways, including a video explaining bodily reactions to dental anxiety and various illustrations.

The website consisted of three modules which focused on (1) DA, (2) dental visits and descriptions of common dental procedures (check-ups, placing a filling, anesthesia, tooth extraction, and root canal treatment), and (3) practical advice on how to manage DA. The academic content within these three modules was mainly based on a Norwegian textbook on oral health psychology [45]. A link to the TADA program was added to the website for those with severe DA/dental phobia.

### 2.2. Study Design, Sample Size and Participants

A quasi-experimental pretest-posttest design was used to evaluate the effectiveness of the developed web-based interventions both in Lithuania and Norway. Sample size calculation, based on a power of 80% and a level of significance of 5%, for detecting a mean of the differences of 4 between pairs, assuming the standard deviation of the differences to be 8, showed 34 participants in each country [46]. In Norway, an invitation to participate in this study was placed on the bulletin boards, information screens, the internal corporate website at UiT, and on the personal Facebook accounts of the co-authors (SSSM and MST). In Lithuania, the invitation was posted on the Facebook account of the website and on the news portal Delfi.lt. In addition, dentists treating patients with dental anxiety informed their patients about the intervention and invited them to participate. Volunteers aged 18–60 years interested in participating contacted the project email address in order to receive further instructions. Before getting access to the website, the participants had to sign an electronic informed consent in Norway and hand-written informed consent in Lithuania. After answering a pretest questionnaire, the participants were invited to use the website actively for two weeks, and a reminder was sent to their email addresses after around one week. After using the website, the participants answered a posttest questionnaire. Outcomes were assessed after two weeks. Participants who completed the posttest questionnaire received around a ten euros value incentive (voucher for a café in Lithuania and cinema tickets in Norway).

### 2.3. Questionnaire

A structured questionnaire in the respective languages was used in Lithuania and Norway. The questionnaires contained the same elements and scales in each country to facilitate comparisons between the countries. The pretest questionnaire inquired about participants’ background characteristics, age, gender, education, urban/rural residency, and presence of DA within close family. It also included the Oral Health Self-Efficacy Scale (OHSES) [47], which consisted of 13 statements with a 5-point Likert scale ranging from disagree to fully agree. The statements were within three specific topics: oral health, oral hygiene, and dental visits. The questionnaire was developed in the Norwegian language, translated/back-translated to Lithuanian language by two independent persons and minor inconsistencies were corrected. DA level was measured using translated versions of the Modified Dental Anxiety Scale (MDAS) [48,49,50,51].

The posttest questionnaire assessed the posttest MDAS score and inquired how many modules participants had visited, if participants had a dental visit during the web-based intervention period, the reason for this visit, and the participant’s experience of the dental visit.

### 2.4. Ethical Considerations

In Norway, approvals were obtained from the Regional Committee for Medical Research Ethics (approval number 86483) and the Norwegian Centre for Research Data (approval number 782570). In Lithuania, approval was obtained from the Lithuanian Biomedical Committee (approval number 2019/10-1162-652). Voluntary participation was based on actively agreeing to the electronic informed consent via Nettskjema-Universitet i Oslo in Norway and signed informed consent in Lithuania.

### 2.5. Statistical Analysis

Statistical analyses were performed in Statistical Package for the Social Sciences (SPSS, Version 28.0, IBM, Armonk, New York, NY, USA). Chi-square and Mann–Whitney U tests were used to compare participants’ characteristics between countries. The Wilcoxon Signed Ranked test was used to compare pretest and posttest MDAS scores stratified by country. Kendall’s tau-b correlation coefficients were used to explore relations between the pretest, posttest and change in OHSE and MDAS scores. OHSE and MDAS change scores were calculated by subtracting the pretest score from the posttest score. A negative OHSE change score indicated a decreased OHSE level post-intervention, while a positive OHSE change score indicated an increased OHSE level post-intervention. The same was true for the MDAS change score: A negative value indicated that levels of DA decreased post-intervention, while a positive value indicated increased DA levels post-intervention. The level of significance was set at *p* < 0.05.

## 3. Results

### 3.1. Participants

In total, 49 participants in Lithuania and 38 in Norway completed the pretest questionnaire. Of these participants, 34 (69%) in Lithuania and 35 (92%) in Norway completed the posttest questionnaires and were thus included in this study.

In Lithuania, a higher proportion of participants who completed the intervention were more educated (bachelor’s degree or more vs. secondary school) and resided in urban areas compared to those that did not complete the intervention. Otherwise, no differences in background characteristics were observed. In Norway, no differences were found between participants who completed the intervention and those who did not (data not shown).

Characteristics of included participants are summarized and compared in Table 3. In Norway, a higher proportion of participants reported having a secondary school education (vs. a bachelor’s degree or more). None of the participants in Lithuania visited all six modules, while in Norway, there were three (9%) participants who did not visit any of the modules. In Lithuania, 11 (32%) participants and in Norway, six (17%) participants reported having dental visits during the intervention; all but one reported a positive experience. Pretest OHSE and MDAS scores were not significantly different between Lithuania and Norway. At the baseline, half of the participants in both countries reported high or severe levels of DA; in Lithuania, 17 (50%) and in Norway, 18 (51%) (Table 4).

### 3.2. Oral Health Self-Efficacy (OHSE)

In total, after two weeks of participating in the web-based intervention, the OHSE score increased for 45 (65%) participants, remained the same for three (4%) participants, and decreased for 21 (30%) of the participants. The posttest OHSE score increased statistically significantly only in Norway; the median OHSE posttest score was 53 (interquartile range (IQR) 10), and the median OHSE pretest score was 52 (IQR 12); Z = 2.573, *p* = 0.01 (Table 4).

### 3.3. Dental Anxiety (DA)

Posttest DA levels decreased for 45 (65%) participants, remained the same for 18 (26%) participants, and increased for six (9%) participants. The median posttest MDAS scores decreased compared to the median pretest MDAS scores in both countries. In Lithuania, the median MDAS pretest score was 14.5 (IQR 8), and the median posttest score was 9.5 (IQR 5.25); Z = −4.246, *p* < 0.001. In Norway, the median MDAS posttest score was 12 (IQR 9), and the median MDAS pretest score was 15 (IQR 7); Z = −3.818, *p* < 0.001 (Table 4). In Lithuania, pretest MDAS score negatively correlated with MDAS change score, indicating that participants with higher DA levels at baseline reported more reduced DA after the web-based intervention (Table 5). 

### 3.4. Correlation between OHSE and DA

Pretest OHSE negatively correlated with the pretest MDAS score and OHSE change score and positively with the posttest OHSE score; these correlations were statistically significant in both countries. Posttest OHSE scores were negatively correlated with posttest MDAS scores only in Lithuanian adults, as well as OHSE change scores with MDAS change scores (Table 5).

## 4. Discussion

The two European countries participating in this pilot study were ranked differently according to the LPI. Therefore, the design and implementation of the web-based interventions were tailor-made and conditioned by the countries’ differences in education, health and living conditions. After two weeks, adult participants who completed a web-based intervention in Lithuania and Norway reported reduced levels of DA. This finding supports the usefulness of web-based interventions for DA in adults in European countries and possibly for other developed countries (probably those that are ranked among the top 80–100 countries, according to the LPI). Therefore Hypothesis 1, which stated that web-based interventions would contribute to reducing participants’ DA, was supported. The results related to OHSE were different: OHSE increased only among Norwegian participants, while Lithuanian participants who reported increased OHSE also reported reduced DA. Hypothesis 2, which stated that since the web-based interventions are conditioned by the culture in which they were designed and implemented, the differences between Norway and Lithuania with regards to how the interventions change the participants’ beliefs about their own ability to cope with their oral health concerns (as measured by OHSE) would be observed, was supported.

Regarding dental treatment, there is some evidence that anxiety appears to be more related to thought processes compared to thought content, which would make self-efficacy unreliable as a means of DA reduction [52]. However, this study investigated a specific type of self-efficacy, namely oral health self-efficacy, and demonstrated that pretest OHSE and pretest MDAS scores were negatively correlated in both counties. This finding is in line with results from the UK and Korea, showing that individuals with higher levels of both general or specific self-efficacy report lower levels of DA [22,24]. However, a recent study from an urban German population found that higher levels of general self-efficacy were not associated with DA but with generalized anxiety [21]. Nevertheless, the results related to oral health self-efficacy in this context indicate that individuals’ thoughts and perceptions about self-confidence and control related to oral health behaviors and procedures are important factors in determining how individuals cope with stressful situations in dentistry [53] and that the intervention might have had some influence over these parameters. Indeed, looking at a recent systematic review related to the effects of non-pharmacological interventions targeting a reduction of DA in patients undergoing third molar extraction, approximately one-third of the interventions appear to either address the preoperative provision of information (i.e., knowledge) or self-efficacy specifically [54].

The results indicated that post-intervention OHSE increased only among the participants in Norway. While OHSE did not significantly increase in Lithuania, a negative correlation between changed levels in OHSE and DA was observed, i.e., if participants reported increased OHSE, they were likely to also report reduced DA. This country-based ambiguity in our findings related to the correlations between OHSE and DA might be partially due to self-selection bias in Lithuania: specifically, that more educated participants completed the web-based intervention. In addition, participants in Lithuania had a lower baseline OHSE compared to participants in Norway, although the difference was not statistically significant. Most likely, the results can be explained by the differences in the countries’ LPI ranking [42]. These results for the relation between OHSE and DA should be applicable to countries within the European context. The relationship between OHSE and DA could be further investigated in different cultures and also in the context of non-European countries accounting for countries’ overall quality of life and human development level, which is captured by the LPI.

The tailor-made web-based intervention design reflected the oral health literacy levels of the participants. Norway had one of the highest Human Development Index rankings in 2020 [55], with inhabitants potentially having high levels of health literacy. Lithuania is one of the least wealthy European Union countries, and an inadequate level of health literacy among Lithuanians has been reported to be higher compared to several other European countries [56]. Therefore in Lithuania, the website constituted six modules, which included full-text popular scientific articles enriched with visual aids, such as figures and videos. In addition, there were suggested homework tasks asking participants to reflect on the newly read text, remember the content of the previous modules, and suggest ways to practically apply the advice. The Lithuanian intervention was arguably more comprehensive compared to the intervention in Norway. In Norway, the web-based intervention was developed to be relatively minimalistic where the website consisted of three modules and included short texts. It is not possible to conclude which aspects of the interventions may have been most effective in reducing DA, as individual parts of the interventions could not be assessed. It should be noted that none of the Lithuanian participants visited all modules on the website, which may be explained by the potentially overwhelming amount of information contained within the modules.

The two websites were different in content, the number of modules, the requirement of homework, etc. Therefore, the comparison between them is limited. Although the web-based intervention was designed to be used by individuals with low or moderate levels of DA, approximately half of the participants in both countries reported high or severe DA, which may have been a limitation as this indicates some form of selection bias. The results showed that participants with higher DA levels at baseline reported more reduced DA in Lithuania. Another limitation of the present study was the design. As this study was not a randomized controlled trial, i.e., lacking a placebo group (e.g., an irrelevant website) or control group (e.g., no website), the current study design could not demonstrate the effectiveness of these tailor-made web-based interventions compared to controls. Furthermore, only short-term outcomes were recorded. Even though the intention was to register outcomes after three months, this proved to be infeasible due to a high dropout rate. Moreover, no outcomes were recorded during real dental visits. Although there were 16 participants who visited a dentist during the intervention period, all of them reported having had a positive experience, except one who reported a neutral experience.

## 5. Conclusions

The present pilot study demonstrated that tailor-made web-based interventions had the potential to reduce dental anxiety levels in two European countries, Lithuania and Norway. These results support the short-term effects of web-based interventions aiming at reducing dental anxiety within the European context. In Lithuania, the participants with higher dental anxiety levels at baseline reported more reduced dental anxiety after the completion of the intervention. Oral health self-efficacy increased among Norwegian adult participants, while Lithuanian participants who reported increased OHSE also reported reduced DA. These results may be explained by the difference in countries’ cultures. Studies with controlled designs assessing long-term outcomes are needed to validate the results of this study and further explore the association between oral health self-efficacy and dental anxiety in different cultures, also outside European countries.

## Figures and Tables

**Table 1:** Advice for participants and key messages presented in each of the modules on web-based intervention in Lithuania and Norway.

Module	Lithuania		Norway	
No	Advice for Participants	Key Messages	Advice for Participants	Key Messages
1	DA is a normal condition. Recognize signs of DA. Get to know dental procedures. Ask for breaks. Agree about signs you can use to stop or start treatment. Try relaxation techniques. Reward yourself after a visit.	DA is a prevalent condition. Recognizing DA signs and learning about causes of DA, as well as self-support techniques, may help to reduce DA.	DA is a normal condition. Tell your dentist about your feelings. Bodily reactions are normal Learn about panic attacks.	Learning about the physiology of fear, recognizing DA symptoms and perceiving them as normal bodily reactions may help to reduce DA.
2	Visit the dentist regularly. Get to know oral diseases. Learn and practice oral disease prevention. Ask your dentist about oral diseases. Reflect about what oral health symptoms you experience.	Learning about and practicing prevention helps to reduce oral health conditions. Learning about oral diseases increases self-confidence when talking to the dentist. Regular check-ups help to avoid complicated procedures.	Get to know dental procedures. Visit the dentist regularly.	Learning about common dental procedures, such as anesthesia, tooth extraction, dental caries, and root canal treatment, as well as terms, instruments, and equipment used in a dental office may help to decrease DA.
3	Visit the dentist regularly. Brush your teeth regularly and use correct products. Brush teeth correctly. Use floss and interdental brushes correctly. Eat healthily.	It is easier to apply preventive measures than to perform treatment. Information about correct use of oral hygiene measures.	Tell the dentist you experience DA. Tell the dentist if you want to be told about procedures or want to be distracted. Agree about signs you can use to stop or start treatment. Ask for breaks. During the treatment, focus on other things. Try relaxation techniques. If you are involved in other forms of therapy, inform the dentist for collaboration with a psychologist or another doctor.	Communication between the dentist and the patient is important. Treatment may be stopped at any time. There are measures that can help if DA increases during treatment.
4	Get to know dental examination procedures. Ask for a break before the second step. If you do not feel well during a professional procedure, show signs to stop, ask for a break or continue next time.	Learning about professional hygiene procedures may help to reduce DA. Dentists could help to improve tooth brushing skills. Good oral hygiene helps to experience no pain during professional hygiene.		
5	Get to know dental caries treatment procedures. Tell your dentist if you are afraid of needles or pain. During treatment, focus on other things.	There are ways to decrease discomfort related to anesthesia or pain. Knowledge about dental caries treatment procedures may reduce DA. Feelings of not having control of the process may be an important cause of DA.		
6	Get to know root canal treatment procedures. Ask for breaks, as root canal treatment can be divided into a few visits. It is normal to experience unusual smells. Focus on treatment steps. If DA increases, use relaxation techniques.	Timely treatment of pulp diseases helps to prevent pain and complicated procedures in the future. Knowledge about root canal treatment may decrease DA. Endodontic treatment may seem scary. Therefore, there is a need to talk about root canal treatment with your dentist.		

**Table 2:** Description of the websites used in the web-based interventions to reduce dental anxiety in Lithuania and Norway.

Website Characteristics	Lithuania	Norway
Website title	(Ne)Bijau “(Not)Afraid”	TannVett “ToothSense”
URL	“https://www.virtualusodontologas.lt/category/nebijau/ (accessed on 13 February 2023)”.	“https://uit.no/research/tannvett (accessed on 13 February 2023)”.
Part of a bigger website	Yes, a subsection of the oral health education website	Yes, related to the university website
Number of modules	6	3
Topics	0. Introduction about the web-based intervention and invitation to participate 1. Dental anxiety and advice 2. Tooth anatomy, etiology of oral diseases and their treatment 3. Preventive measures and personal oral hygiene 4. Professional oral hygiene, including check-ups, advice during the procedure 5. Dental visit: anesthesia, rubber-dam, caries removal, filling, advice during the procedure 6. Root canal treatment	0. Introduction of the website 1. Dental anxiety 2. Dental visit: terms, instruments, check-up, filling including rubber-dam, anesthesia, extraction, root canal treatment 3. Advice
Presentation of modules	Full-length articles, pictures, schematic illustrations, and links to other online sources (for example, videos)	Short text, pictures, pop-up subsections, 1 video
Homework	Yes, at the end of each module. Reflection on information provided in the module and repetition of other modules and/or advice	No
Advised intervals between modules	Yes, not more than one module per day	No
Information for those with severe dental anxiety	No	Yes, link out to the TADA program

URL—uniform resource locator. TADA program—dental treatment offered for persons exposed to torture, abuse and dental phobia (“Tilrettelagt tannbehandling til personer utsatt for Tortur eller Overgrep, eller som har Odontofobi”).

**Table 3:** Characteristics of the participants included in web-based intervention to reduce dental anxiety in Lithuania and Norway.

Characteristics of Participants	Lithuania *n* (%)	Norway *n* (%)
Sex	34 (100)	35 (100)
female	25 (74)	31 (89)
male	9 (26)	4 (11)
Age ^i^	34 (100)	35 (100)
mean (SD)	40.7 (14.5)	32.7 (11.6)
median (IQR)	39.5 (28.25)	28 (23)
Education ^ii^	34 (100)	35 (100)
secondary	6 (18)	16 (46)
bachelor’s degree	18 (53)	8 (23)
master’s degree or more	10 (29)	11 (31)
Residency	34 (100)	35 (100)
urban	22 (65)	23 (66)
periurban/rural	12 (35)	12 (34)
Family history of dental anxiety ^ii^	34 (100)	35 (100)
yes	16 (47)	13 (37)
no	2 (6)	15 (43)
I do not know	16 (47)	7 (20)
Number of modules visited *	34 (100)	35 (100)
0	0 (0)	3 (9)
1	1 (3)	4 (11)
2	5 (15)	3 (9)
3	3 (9)	25 (71)
4	25 (73)	N/A
Dental visit during the intervention	34 (100)	35 (100)
yes	11 (32)	6 (17)
no	23 (68)	29 (83)
Dental visit reason	11 (100)	6 (100)
acute	1 (9)	2 (33)
planned	10 (91)	4 (67)
Experience during the dental visit	11 (100)	6 (100)
negative	0 (0)	0 (0)
neutral	1 (9)	0 (0)
positive	10 (91)	6 (100)

^i^ *p* < 0.05 according to Mann–Whitney U test comparison between countries; ^ii^ *p* < 0.05 according to Chi-square test comparison between countries; N/A—not applicable; * not compared due to the different number of modules in each country. The maximum number of modules in Lithuania was six, and in Norway—three.

**Table 4:** Comparison of pretest and posttest MDAS and OHSE scores among adults in Lithuania and Norway.

Scores of Participants	Lithuania *n* = 34 (%)	Norway *n* = 35 (%)
MDAS pretest ^iii^	34 (100)	35 (100)
Low DA (MDAS score 0–10)	9 (27)	7 (20)
Moderate DA (MDAS score 11–14)	8 (23)	10 (28)
High DA (MDAS score 15–18)	10 (29)	8 (24)
Severe DA (phobic) (MDAS score 19–25)	7 (21)	10 (28)
Mean pretest MDAS (SD)/ median pretest MDAS (IQR)	14.7 (5.1)/ 14.5 (8)	15.0 (5.3)/ 15 (7)
MDAS posttest	34 (100)	35 (100)
Low DA (MDAS score 0–10)	23 (67)	13 (37)
Moderate DA (MDAS score 11–14)	4 (12)	9 (26)
High DA (MDAS score 15–18)	6 (18)	7 (20)
Severe DA (phobic) (MDAS score 19–25)	1 (3)	6 (17)
Mean posttest MDAS (SD)/ median posttest MDAS (IQR)	10.4 (4.4)/ 9.5 (5.25)	13.4 (5.1)/ 12 (9)
OHSE pretest ^iv^		
mean (SD)/ median (IQR)	47.8 (6.4)/ 48 (8.5)	50.9 (8.8)/ 52 (12)
OHSE posttest		
mean (SD)/ median (IQR)	50.1 (8.1)/ 51 (9.75)	53.1 (8.3)/ 53 (10)

^iii^ *p* < 0.05 according to Wilcoxon Signed Ranks test, comparison between MDAS pretest and posttest scores in Lithuania and in Norway; ^iv^ *p* < 0.05 according to Wilcoxon Signed Ranks test, comparison between OHSE pretest and posttest scores only in Norway; MDAS—Modified Dental Anxiety Scale; OHSE—Oral Health Self-Efficacy Scale.

**Table 5:** Kendall’s tau-b correlation coefficient, *p*-value between pretest OHSE and MDAS scores, posttest OHSE and MDAS score, OHSE change score, and MDAS change score.

	Pretest MDAS Score	Posttest OHSE Score	Posttest MDAS Score	OHSE Change Score	MDAS Change Score
**Pretest OHSE score**	*−0.341*; **−0.241** *0.006*; **0.049**	*0.292*; **0.683** *0.018*; **<0.001**	*−0.208*; **−0.138** *0.099*; **0.259**	*−0.257*; **−0.351** *0.037*; **0.004**	*0.209*; **0.164** *0.101*; **0.196**
**Pretest MDAS score**		*−0.052*; **−0.238** *0.676*; **0.052**	*0.456*; **0.807** *<0.001*; **<0.001**	*0.208*; **0.057** *0.095*; **0.646**	*−0.452*; **−0.231** *<0.001*; **0.071**
**Posttest OHSE score**			*−0.291*; **−0.179** *0.021*; **0.145**	*0.480*; **0.000** *<0.001*; **1.000**	*−0.238*; **−0.113** *0.062*; **0.376**
**Posttest MDAS score**				*−0.167*; **−0.020** *0.187*; **0.875**	*0.164*; **0.022** *0.208*; **0.861**
**OHSE change score**					*−0.460*; **−0.177** *<0.001*; **0.171**

OHSE—Oral Health Self-Efficacy scale; MDAS—Modified Dental Anxiety Scale; Values from *Lithuania (in italics)*; Values from **Norway (in bold)**.

## Data Availability

The data presented in this study are available on request from the corresponding authors. The data are not publicly available due to sensitivity.

## References

[1] Crego A., Carrillo-Diaz M., Armfield J.M., Romero M. (2014). From public mental health to community oral health: The impact of dental anxiety and fear on dental status. Front. Public Health.

[2] Slabšinskienė E., Kavaliauskienė A., Žemaitienė M., Vasiliauskienė I., Zaborskis A. (2021). Dental fear and associated factors among children and adolescents. A school-based study in Lithuania. Int. J. Environ. Res. Public Health.

[3] McGrath C., Bedi R. (2003). The association between dental anxiety and oral health-related quality of life in Britain. Community Dent. Oral Epidemiol..

[4] Eitner S., Wichmann M., Paulsen A., Holst S. (2006). Dental anxiety—An epidemiological study on its clinical correlation and effects on oral health. J. Oral Rehabil..

[5] Vermaire J.H., De Jongh A., Aartman I.H.A. (2008). Dental anxiety and quality of life: The effect of dental treatment. Community Dent. Oral Epidemiol..

[6] Alharbi A., Freeman R., Humphris G. (2021). Dental anxiety, child-oral health related quality of life and self-esteem in children and adolescents: A systematic review and meta-analysis. Community Dent. Health.

[7] Silveira E.R., Cademartori M.G., Schuch H.S., Armfield J.A., Demarco F.F. (2021). Estimated prevalence of dental fear in adults: A systematic review and meta-analysis. J. Dent..

[8] Raadal M., Skaret E., Öst L.G., Skaret E. (2013). Background description and epidemiology. Cognitive Behaviour Therapy for Dental Phobia and Anxiety.

[9] Svensson L., Hakeberg M., Boman U.W. (2016). Dental anxiety, concomitant factors and change in prevalence over 50 years. Community Dent. Health.

[10] Locker D., Liddell A., Shapiro D. (1999). Diagnostic categories of dental anxiety: A population-based study. Behav. Res. Ther..

[11] Halonen H., Nissinen J., Lehtiniemi H., Salo T., Riipinen P., Miettunen J. (2018). The association between dental anxiety and psychiatric disorders and symptoms: A systematic review. Clin. Pract. Epidemiol. Ment. Health CP EMH.

[12] Minja I.K., Kahabuka F.K., Kocabaşoğlu N., Çağlayan R.H.B. (2019). Dental anxiety and its consequences to oral health care attendance and delivery. Anxiety Disorders—From Childhood to Adulthood.

[13] Armfield J.M., Stewart J.F., Spencer A.J. (2007). The vicious cycle of dental fear: Exploring the interplay between oral health, service utilization and dental fear. BMC Oral Health.

[14] Bryne E., Hean S.C.P.D., Evensen K.B., Bull V.H. (2022). Exploring the contexts, mechanisms and outcomes of a torture, abuse and dental anxiety service in Norway: A realist evaluation. BMC Health Serv. Res..

[15] Jankauskiene B., Virtanen J.I., Kubilius R., Narbutaite J. (2013). Treatment under dental general anesthesia among children younger than 6 years in Lithuania. Medicina.

[16] Corrigan P.W., Druss B.G., Perlick D.A. (2014). The impact of mental illness stigma on seeking and participating in mental health care. Psychol. Sci. Public Interest.

[17] St-Louis L., Robichaud-Ekstrand S. (2003). Knowledge level and coping strategies according to coagulation levels in older persons with atrial fibrillation. Nurs. Health Sci..

[18] Chen Y.-Y., Lee M.-C., Wu S.-F.V., Liu Y.-M., Chen H.-M. (2022). Disease knowledge, self-efficacy, and quality of life in patient with hypertensive nephropathy. Clin. Nurs. Res..

[19] Chuang L.-M., Wu S.-F.V., Lee M.-C., Lin L.-J., Liang S.-Y., Lai P.-C., Kao M.-C. (2021). The effects of knowledge and self-management of patients with early-stage chronic kidney disease: Self-efficacy is a mediator. Jpn. J. Nurs. Sci..

[20] Bandura A. (1988). Organisational applications of social cognitive theory. Aust. J. Manag..

[21] Valdes-Stauber J., Hummel K. (2021). The relationship between dental anxiety and other kinds of anxiety: A naturalistic, cross-sectional and comparative study. BMC Psychol..

[22] Bae S. (2009). Relationship between dental anxiety and self-efficacy that patients feel while dental hygienist conduct scaling. J. Korean Soc. Dent. Hyg..

[23] Skaret E., Kvale G., Raadal M. (2003). General self-efficacy, dental anxiety and multiple fears among 20-year-olds in Norway. Scand. J. Psychol..

[24] Kent G. (1987). Self-efficacious control over reported physiological, cognitive and behavioural symptoms of dental anxiety. Behav. Res. Ther..

[25] Powell J., Clarke A. (2002). The WWW of the World Wide Web: Who, what, and why?. J. Med. Internet Res..

[26] Chestnutt I.G., Reynolds K. (2006). Perceptions of how the Internet has impacted on dentistry. Br. Dent. J..

[27] Wynn R., Gabarron E., Johnsen J.-A.K., Traver V. (2020). Special issue on e-health services. Int. J. Environ. Res. Public Health.

[28] Tiwari T., Diep V., Tranby E., Thakkar-Samtani M., Frantsve-Hawley J. (2022). Dentist perceptions about the value of teledentistry. BMC Oral Health.

[29] AlKlayb S.A., Assery M.K., AlQahtani A., AlAnazi M., Pani S.C. (2017). Comparison of the effectiveness of a mobile phone-based education program in educating mothers as oral health providers in two regions of Saudi Arabia. J. Int. Soc. Prev. Community Dent..

[30] Marino R.J., Marwaha P., Barrow S.Y. (2016). Web-based oral health promotion program for older adults: Development and preliminary evaluation. Int. J. Med. Inf..

[31] World Health Organization Executive Board Resolution: EB 148.R1. https://apps.who.int/gb/ebwha/pdf_files/EB148/B148_R1-en.pdf.

[32] World Health Organization Draft Global Oral Health Action Plan (2023–2030). WHO Discussion Paper, Version Dated 12 August 2022. https://cdn.who.int/media/docs/default-source/ncds/mnd/eb152-draft-global-oral-health-action-plan.pdf?sfvrsn=ecce482e_4.

[33] Eijlers R., Utens E., Staals L.M., de Nijs P.F.A., Berghmans J.M., Wijnen R.M.H., Hillegers M.H.J., Dierckx B., Legerstee J.S. (2019). Systematic review and meta-analysis of virtual reality in pediatrics: Effects on pain and anxiety. Anesth. Analg..

[34] Kim J., Chiesa N., Raazi M., Wright K.D. (2019). A systematic review of technology-based preoperative preparation interventions for child and parent anxiety. Can. J. Anesth..

[35] Gujjar K.R., van Wijk A., Kumar R., de Jongh A. (2019). Are technology-based interventions effective in reducing dental anxiety in children and adults? A systematic review. J. Evid. Based Dent. Pract..

[36] Cunningham A., McPolin O., Fallis R., Coyle C., Best P., McKenna G. (2021). A systematic review of the use of virtual reality or dental smartphone applications as interventions for management of paediatric dental anxiety. BMC Oral Health.

[37] Porritt J., Rodd H., Morgan A., Williams C., Gupta E., Kirby J., Creswell C., Newton T., Stevens K., Baker S. (2017). Development and testing of a cognitive behavioral therapy resource for children’s dental anxiety. JDR Clin. Trans. Res..

[38] Lahti S., Suominen A., Freeman R., Lähteenoja T., Humphris G. (2020). Virtual reality relaxation to decrease dental anxiety: Immediate effect randomized clinical trial. JDR Clin. Trans. Res..

[39] Mellish V. (2019). Study of online information for anxious patients requiring dental extractions. Br. Dent. J..

[40] Lin X., Li S., Yang S., Zheng X. (2022). Preoperative online intervention to dental anxiety in patients with transcrestal sinus floor elevation. Quintessence Int..

[41] Zahedi F.M., Van Pelt W.V., Song J.A., Beer D.F. (2001). Conceptual framework for international Web design. Writing and Speaking in the Technology Professions.

[42] Legatum Institute 2019 Legatum Prosperity Index™. https://li.com/reports/2019-legatum-prosperity-index/.

[43] Appukuttan D.P. (2016). Strategies to manage patients with dental anxiety and dental phobia: Literature review. Clin. Cosmet. Investig. Dent..

[44] Armfield J.M., Heaton L.J. (2013). Management of fear and anxiety in the dental clinic: A review. Aust. Dent. J..

[45] Willumsen T., Myran L., Lein J.P.Å. (2018). Odontologisk Psykologi.

[46] Dhand N.K., Khatkar M.S. Statulator: An Online Statistical Calculator. Sample Size Calculator for Comparing Two Paired Means. http://statulator.com/SampleSize/ss2PM.html.

[47] Steinvik L., Johnsen J.-A.K., Svartdal F. The measurement of oral health self-efficacy and its relation to procrastination tendencies. Proceedings of the CED-IADR/NOF Oral Health Research Congress.

[48] Humphris G.M., Morrison T., Lindsay S.J. (1995). The Modified Dental Anxiety Scale: Validation and United Kingdom norms. Community Dent. Health.

[49] Haugejorden O., Klock K.S. (2000). Avoidance of dental visits: The predictive validity of three dental anxiety scales. Acta Odontol. Scand..

[50] Brukiene V., Aleksejuniene J., Balciuniene I. (2006). Is dental treatment experience related to dental anxiety? A cross-sectional study in Lithuanian adolescents. Stomatologija.

[51] Humphris G. Modified Dental Anxiety Scale Translations 2022. https://www.st-andrews.ac.uk/dentalanxiety/scaletranslations.

[52] Kent G., Gibbons R. (1987). Self-efficacy and the control of anxious cognitions. J. Behav. Ther. Exp. Psychiatry.

[53] Litt M.D., Nye C., Shafer D. (1993). Coping with oral surgery by self-efficacy enhancement and perceptions of control. J. Dent. Res..

[54] Wong N.S.M., Yeung A.W.K., Li K.Y., McGrath C.P., Leung Y.Y. (2022). Non-pharmacological interventions for reducing fear and anxiety in patients undergoing third molar extraction under local anesthesia: Systematic Review and Meta-Analysis. Int. J. Environ. Res. Public Health.

[55] (2022). United Nations Development Program Human Development Index. https://hdr.undp.org/data-center/human-development-index#/indicies/HDI.

[56] Gatulytė I., Verdiņa V., Vārpiņa Z., Lublóy Á. (2022). Level of health literacy in Latvia and Lithuania: A population-based study. Arch. Public Health.

